# Extinction of Heroin Seeking Does Not Require the Infralimbic Cortex or Its Projections to the Nucleus Accumbens Shell or Amygdala

**DOI:** 10.1111/adb.70092

**Published:** 2025-10-21

**Authors:** Matthew S. McGregor, Kelle E. Nett, Subhash C. Gupta, John A. Wemmie, Ryan T. LaLumiere

**Affiliations:** ^1^ Interdisciplinary Graduate Program in Neuroscience University of Iowa Iowa City Iowa USA; ^2^ Department of Psychiatry University of Iowa Iowa City Iowa USA; ^3^ Department of Veterans Affairs Medical Center Iowa City Iowa USA; ^4^ Department of Molecular Physiology and Biophysics University of Iowa Iowa City Iowa USA; ^5^ Medical Scientist Training Program University of Iowa Iowa City Iowa USA; ^6^ Department of Neurosurgery University of Iowa Iowa City Iowa USA; ^7^ Iowa Neuroscience Institute University of Iowa Iowa City Iowa USA; ^8^ Department of Psychological and Brain Sciences University of Iowa Iowa City Iowa USA

**Keywords:** amygdala, extinction, heroin, infralimbic, nucleus accumbens

## Abstract

Evidence indicates that the activity of the infralimbic cortex (IL), as well as its projections to the nucleus accumbens shell (NAshell) and amygdala, following an unreinforced lever press is critical for cocaine extinction learning and retention. It is unclear whether the same neural circuitry is involved in extinction encoding for other classes of addictive drugs, including opioids. In this study, we used a behaviour‐controlled optogenetic approach in female and male Sprague–Dawley rats to examine the role of the IL and its projections in the extinction of heroin seeking. Rats first underwent 12+ days of 6‐ or 3‐h heroin self‐administration sessions, followed by 12 days of extinction training. Optogenetic inhibition of the IL, IL‐NAshell or IL‐amygdala pathway was given for 20 s immediately following an unreinforced lever press during the first 5 days of extinction. Unlike cocaine extinction, these manipulations had no effect on lever pressing during extinction training, nor on the retention of extinction learning, as assessed during the subsequent 7 days of extinction without optogenetic inhibition. These results suggest that the extinction of heroin seeking does not involve the same infralimbic mechanisms that are critical for the extinction of cocaine seeking. Although extinction learning did not differ by sex, analyses of self‐administration data revealed that females self‐administered more heroin than males in 3 h, but not 6 h, self‐administration sessions, indicating session length‐dependent sex differences in heroin taking.

## Graphical abstract

This study found that optogenetic inhibition of the infralimbic cortex, or infralimbic projections to the nucleus accumbens shell or amygdala, following unreinforced lever presses had no effect on extinction of heroin seeking in rats. Similar manipulations impaired cocaine extinction learning in the authors’ prior work, indicating that the neural mechanisms of extinction learning are drug‐type dependent.

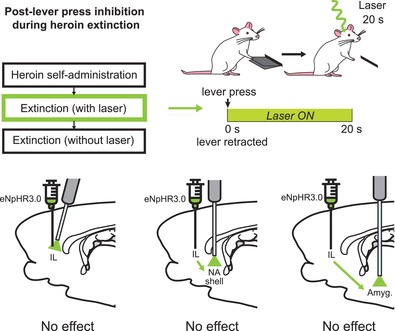

## 1 Introduction

Considerable evidence suggests that the rat infralimbic cortex (IL) regulates the extinction and inhibition of cocaine seeking [1]. Prior work from our laboratory found that optogenetic inhibition of the IL [2] or its projections to the nucleus accumbens shell (NAshell) or amygdala [3], in the 20 s immediately following an unreinforced lever press, impaired the extinction of cocaine seeking. These findings support the idea that this period is critical for encoding a prediction error following an unrewarded response. However, it is unclear whether these mechanisms are involved in extinction learning for other classes of addictive drugs, including opioids.

Several prior studies support a role for the IL in suppressing opioid seeking, consistent with work using cocaine and other addictive drugs. Evidence suggests that disrupting IL activity increases expression of morphine‐ and heroin‐induced conditioned place preference [4, 5] and that chemogenetic inactivation of the IL‐NAshell pathway increases cued heroin seeking during acute withdrawal [6]. This is consistent with an inhibitory role for this pathway in cocaine and alcohol seeking [3, 7, 8]. Correspondingly, evidence indicates that enhancing IL activity via AMPA receptor activation facilitates extinction and attenuates cued reinstatement of heroin seeking [9, 10], suggesting IL involvement in both extinction learning and the ongoing inhibition of opioid seeking. Although no prior work has investigated IL projections to the amygdala in opioid seeking, the IL innervates several amygdala targets, including the basolateral amygdala (BLA), the capsular cells of the central nucleus (CLC) and the medial cluster of intercalated cells (mITC), all of which are implicated in extinction of conditioned fear [11, 12, 13, 14]. Indeed, evidence indicates that IL projections to the amygdala are important for extinction of conditioned fear [15] and cocaine seeking [3], suggesting a common mechanism for extinction across distinct behavioural paradigms [16].

In contrast, other evidence indicates that reversible IL inactivation reduces heroin‐primed, cued and contextual reinstatement of heroin seeking after extinction [17], suggesting that IL activity *promotes* opioid seeking under some circumstances. Peters et al. [18] proposed that competing neuronal ensembles within the IL and with distinct outputs may account for these disparate findings; however, evidence indicates that disconnection of the IL–NAshell pathway also reduces contextual reinstatement of heroin seeking [19]. These findings reveal potentially divergent regulation of heroin vs. cocaine seeking after extinction by the same IL circuitry, raising questions about their role in encoding heroin extinction learning.

To address these issues, the present study used behaviour‐controlled optogenetic inhibition of the IL or its projections to the NAshell or amygdala immediately following an unreinforced lever press during extinction of heroin seeking in female and male rats. In contrast to our prior work with extinction of cocaine seeking [2, 3], the results suggest that these infralimbic mechanisms are not necessary for the encoding of heroin extinction learning.

## 2 Materials and Methods

### 2.1 Subjects

Female and male Sprague–Dawley rats (200–225 and 225–250 g, respectively, at the time of the first surgery; Envigo; *n* = 80) were used in this study. All rats were single‐housed in a temperature‐controlled environment under a 12‐h light/dark cycle (light on at 06:00) and allowed to acclimate to the vivarium for at least 2 days before surgery. All procedures followed the National Institutes of Health guidelines for the care and use of laboratory animals and were approved by the University of Iowa Institutional Animal Care and Use Committee.

### 2.2 Surgery

All rats underwent same‐day cranial virus and optical fibre implantation surgeries, followed 2 weeks later by an intrajugular catheter implantation surgery. In all surgeries, rats were anaesthetized with 3%–5% isoflurane. Meloxicam (2 mg/kg, s.c.) was administered as an analgesic before surgery as well as 24 h after surgery. Rats also received sterile saline (3 mL, s.c.) after surgery for rehydration.

For cranial surgeries, rats were placed in a small animal stereotax (Kopf Instruments) and injected with virus (AAV‐CaMKIIα‐eNpHR3.0‐eYFP or AAV‐CaMKIIα‐eYFP; 0.3 μL) bilaterally into the IL (AP: +2.7 mm and ML: +/−0.6 mm, from Bregma; DV: −5.2 mm from skull surface) through double‐barreled 33 gauge injectors (1.2 mm centre‐to‐centre distance; Plastics One) at a rate of 0.1 μL/min. Injectors were left in place for 7 min to allow diffusion of the virus. Jeweller's screws were then affixed to the skull surface. Optical fibres were then implanted bilaterally targeting the IL (AP + 2.7 mm and ML +/−1.4 mm, at a 10° inward angle, from Bregma; DV −4.8 mm from skull surface), NAshell (AP + 1.2 mm and ML +/−2.4 mm at a 10° inward angle, from Bregma; DV −7.2 mm from skull surface) or amygdala (AP −2.5 mm and ML +/−5.3 mm, at a 5° inward angle, from Bregma; DV −7.0 mm from skull surface) and secured with dental cement. Optical fibre implants were made in‐house by gluing optical fibres (0.50 numerical aperture, Ø200‐μm core; ThorLabs) into multimode stainless alloy ferrules (10.5 mm length, Ø 2.5‐mm outer diameter, 230‐μm bore; Precision Fiber Products), and the externalized end of the ferrule was polished using lapping sheets with decreasing grit (5–0.3 μm; ThorLabs). Dust caps were maintained on the externalized end of the ferrule throughout the experiments. Virus was allowed to transduce for at least 5 weeks before optogenetic manipulations occurred.

For catheter implantation, a rounded tip jugular vein catheter (SAI Infusion Technologies) with suture beads 2.6 and 3.0 cm (females) or 3.0 and 3.5 cm (males) from the rounded tip was inserted into the right jugular vein. The opposite end of the catheter was externalized between the shoulder blades and connected to a harness with a 22‐gauge guide cannula, which was used for heroin delivery. Catheters were flushed 6 days/week with 0.1 mL of heparinized saline and glycerol to ensure catheter patency. Rats received antibiotics (Baytril, 2.5 mg/kg, s.c.) the day of catheter implantation and for 12 days following surgery. Rats were allowed to recover from surgery for 5 days before beginning heroin self‐administration.

### 2.3 Optical Illumination

During sessions in which rats received optical illumination, rats were connected to a laser (300 mW, 561 nm; OEM Laser Systems) as previously described [2]. Power output was adjusted to ~10 mW at the fibre tip, based on previous work [2, 3, 20]. Upon active lever press, a Master‐8 Stimulator (A.M.P.I.) triggered 20 s of continuous laser illumination.

### 2.4 Heroin Self‐Administration

Rats self‐administered heroin 6 days/week in standard operant conditioning chambers (Med Associates), housed within sound‐attenuating chambers and equipped with two retractable levers. Cue lights were located directly above both levers, and a 4500‐Hz Sonalert pure tone generator module was positioned above the right lever. A 6‐W house light on the opposite wall of the operant chamber was illuminated throughout the training sessions. Heroin (kindly provided by the National Institute on Drug Abuse) was dissolved in 0.9% sterile saline. A concentration of 1.0‐mg/mL heroin was used for the first 2 days of self‐administration in both sexes, followed by concentrations of 0.34‐mg/mL heroin for females and 0.45 mg/mL for males for all subsequent days of self‐administration. These concentrations were chosen to produce a dose of approximately 0.075‐mg‐heroin/kg‐body weight per 50‐μL infusion for both sexes.

Although final self‐administration procedures differed between Experiment 1 and Experiments 2 and 3, rats in all experiments began with FR1 heroin self‐administration. During these sessions, a press on the right (active) lever resulted in a 50‐μL heroin infusion and a 5‐s presentation of light and tone cues above the active lever. A 20‐s timeout period followed each infusion, during which active lever presses were recorded but had no scheduled consequence. A press on the left (inactive) lever had no consequence. Rats self‐administered heroin in either 6 h (Experiment 1) or 3 h (Experiments 2 and 3) FR1 sessions for at least 4 days (≥ 2 days on the lower dose) before moving to experiment‐specific self‐administration procedures described in Section 2.6.

### 2.5 Extinction

Rats began extinction training the day after completing self‐administration. Although lever availability during extinction differed between Experiment 1 and Experiments 2 and 3 (described in Section 2.6), the immediate consequence of an active lever press was identical across experiments. In the first 5 days of 2‐h (Experiment 1) or 30‐min (Experiments 2 and 3) extinction sessions, each active lever press resulted in active lever retraction and 20 s of laser illumination. The lever was retracted in this manner so that rats could not press the active lever during laser illumination. Following these extinction sessions with laser illumination, rats underwent 7 days of 2‐ (Experiment 1) or 3‐h (Experiments 2 and 3) extinction sessions wherein an active lever press resulted in immediate active lever retraction but no laser illumination. The extinction data from these 7 days served as an index of retention of extinction learning from the sessions with laser illumination.

### 2.6 Experimental Design—Experiment 1: IL Inhibition Following an Unreinforced Lever Press During Extinction Training

Experiment 1 examined whether postlever press IL activity is necessary for the extinction of heroin seeking. Here, a viral vector containing either the inhibitory opsin eNpHR3.0 or an eYFP control was injected into the IL, and optical fibres were implanted above the IL. In our initial experiments, because we expected IL inhibition to alter the extinction of heroin seeking, we used procedures similar to those we used previously [21] for future electrophysiological studies. Thus, in Experiment 1, rats first learned to self‐administer heroin in 6‐h FR1 sessions, according to the procedures described above. Following at least 4 days of FR1 self‐administration, with ≥ 20 infusions on the final day, rats proceeded with a trial‐based design for self‐administration and extinction procedures, akin to those used previously [21]. During trial‐based sessions, levers were only available during discrete 90‐s trials. An active lever press resulted in a 50‐μL heroin infusion and 5‐s presentation of light and tone cues above the active lever, along with retraction of both levers and the start of an inter‐trial interval (ITI). The length of the ITI was between 11 and 12 min, pseudorandomly selected and an average of 11.5 min long, based on the average interval between infusions during a standard FR1 session. If the rat did not press the active lever before the end of a 90 s trial, both levers retracted, and the ITI began. Rats self‐administered heroin in trial‐based sessions for at least 3 days, with active lever presses in ≥ 85% of trials on average for the final 3 days. All rats in this experiment completed at least 12 days of self‐administration in total.

After completing self‐administration, rats proceeded with 2‐h trial‐based extinction sessions, wherein levers were only available during discrete 30‐s trials. An active lever press during a trial resulted in retraction of both levers and the start of a 2–3‐min ITI, along with the consequences described in Section 2.5. The shortened ITI was chosen to allow for more heroin‐seeking responses during the shortened extinction session.

### 2.7 Experiment Design—Experiment 2: IL‐NAshell Pathway Inhibition Following an Unreinforced Lever Press During Extinction Training

Experiment 2 examined whether postlever press activity in the IL‐NAshell pathway is necessary for the extinction of heroin seeking. Here, a viral vector containing either eNpHR3.0 or an eYFP control was injected into the IL, and optical fibres were implanted above IL terminals in the NAshell. Because of an unexpected null effect in Experiment 1 that differed from our findings with the extinction of cocaine seeking [2], and the possibility that this discrepancy was due to the new long access, trial‐based experimental design rather than drug type, we used 3‐h access, FR1 heroin self‐administration procedures in Experiment 2 that more closely approximate our prior work with cocaine [3].

Rats first learned to self‐administer heroin in 3‐h FR1 sessions, as previously described. Following at least 4 days of FR1 self‐administration, with ≥ 8 infusions on the final day, rats proceeded with 3‐h self‐administration with lever retraction. In these sessions, an active lever press resulted in a 50‐μL heroin infusion, 5‐s presentation of light and tone cues above the active lever and 20‐s retraction of the active lever, but levers were otherwise available throughout the duration of the session. The active lever was retracted in this manner to familiarize the rat with lever retraction procedures that would occur during extinction. Rats completed at least 3 days of self‐administration with lever retraction, with ≥ 10 heroin infusions on average for the final 3 days. All rats in this experiment completed at least 12 days of self‐administration in total.

After completing self‐administration, rats proceeded with 30‐min extinction sessions with laser illumination for 5 days and then 3‐h sessions without laser illumination for 7 days. This design more closely approximates our prior work with cocaine [3] and was chosen to reduce the amount of extinction learning that occurs during each 30‐min extinction session, thereby enabling the 3‐h (full‐length) sessions to better serve as a measure of retention of extinction learning. In both sets of sessions, levers were available throughout the session except for the 20 s following an unreinforced lever press, during which the active lever was retracted.

### 2.8 Experiment Design—Experiment 3: IL‐Amygdala Pathway Inhibition Following an Unreinforced Lever Press During Extinction Training

Experiment 3 examined whether postlever press activity in the IL‐amygdala pathway is necessary for the extinction of heroin seeking. Here, a viral vector containing either eNpHR3.0 or an eYFP control was injected into the IL, and optical fibres were implanted above IL terminals in the CLC. Because light diffusion from the optical fibres might also have reached IL terminals in the nearby BLA and mITC, this target is referred to as ‘amygdala’ throughout. Procedures were otherwise identical to those used in Experiment 2.

### 2.9 Histology

After the conclusion of behaviour, rats were overdosed with sodium pentobarbital (100 mg/kg, i.p.) and transcardially perfused with 60 mL of PBS (pH 7.4), followed by 60 mL of 4% paraformaldehyde in PBS. Brains were stored in 4% paraformaldehyde for 48 h before sectioning. Brains were coronally sectioned (75 μm) and mounted on gelatin‐coated slides to be stained with Cresyl violet or viewed under a fluorescent microscope. Optical fibre termination points were visualized on Cresyl violet‐stained sections under a light microscope according to the Paxinos and Watson atlas [22]. Viral expression in the IL, NAshell and amygdala was verified with fluorescent microscopy. Rats with misplaced virus expression or optic fibre termination points were excluded from analysis.

### 2.10 Electrophysiological Verification

Because of the unexpected null effects in the present work that differed from our findings with the extinction of cocaine seeking, which used eArchT3.0 to inhibit IL neurons [2, 3], we conducted an electrophysiological experiment to confirm functional eNpHR3.0 expression in ex vivo brain slices. Two rats were injected with AAV‐CaMKIIα‐eNpHR3.0‐eYFP into the bilateral IL as described above. Five weeks after surgery, rats were anaesthetized with isoflurane and decapitated, and coronal IL slices (300 μm) were prepared in ice‐cold sucrose‐based cutting solution. Slices were incubated in artificial cerebrospinal fluid at 32°C for at least 1 h before recording.

Whole‐cell current‐clamp recordings were performed from eYFP^+^ pyramidal neurons in the IL using borosilicate pipettes (3–5 MΩ) filled with K‐gluconate‐based internal solution. Neurons were held in current‐clamp mode to assess excitability. After a stable baseline, 620‐nm LED light was applied for various time periods using an LD‐1 Single Channel LED Driver (PlexBright), and changes in firing rate were recorded. Subsequent analysis was performed in Clampfit software (Axon).

### 2.11 Statistical Analysis

In Experiment 1, an unpaired between‐subjects *t*‐test was used to determine whether the percent of trials with an active lever press averaged across the final 3 days of self‐administration differed between eNpHR3.0 and eYFP groups. For all experiments, lever presses and heroin infusions during the final 3 days of self‐administration were analysed using two‐way, repeated measures ANOVA with day as the within‐subjects variable and group (eNpHR3.0 vs. eYFP) as the between‐subjects variable. The same analysis was also used to analyse active lever presses during the 5 days of extinction with laser illumination, as well as the following 7 days of extinction without laser illumination. Each test was also run separately for females and males as a preliminary analysis to identify potential areas in which differences may emerge. Additionally, because others have reported differences in heroin self‐administration measures between sexes [23, 24, 25], data from the final 3 days of heroin self‐administration were collapsed across groups to identify potential sex differences. Heroin infusions and bodyweight‐adjusted heroin intake were analysed using two‐way ANOVA with day as the within‐subjects variable and sex as the between‐subjects variable. In all analyses, the Greenhouse–Geisser correction was used if the assumption of sphericity was violated. *P*‐values < 0.05 were considered significant for all analyses. All measures were expressed as mean ± SEM. All data were analysed using GraphPad Prism 10.0.2.

## 3 Results

### 3.1 Experiment 1

**FIGURE 1:**
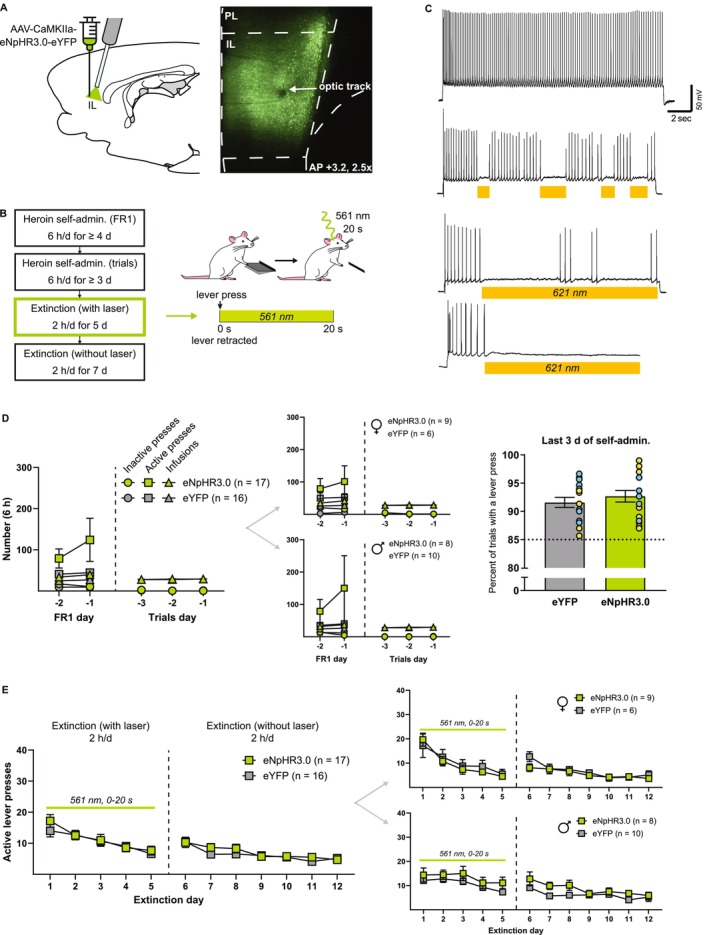
No effect of postlever press IL inhibition on heroin extinction learning. (A) Left, a viral vector containing either the inhibitory opsin eNpHR3.0 or an eYFP control was injected into the IL, and optical fibres were implanted above the IL. Right, representative fluorescent image depicting virus expression and optical fibre termination in the IL. (B) Experimental timeline. After recovering from surgery, rats were trained on 6‐h FR1 heroin self‐administration, followed by 6‐h trial‐based sessions wherein levers were only available during discrete trials and an active lever press resulted in a heroin infusion, light and tone cues and retraction of both levers. Self‐administration was followed by 5 days of 2‐h trial‐based extinction sessions with laser illumination, wherein each active lever press resulted in retraction of both levers and 20‐s laser illumination. Rats then underwent 7 days of 2‐h trial‐based extinction sessions without laser illumination to assess the retention of extinction learning. (C) Representative images of a whole‐cell current‐clamp recording from an IL neuron expressing eNpHR3.0. Illumination with LED light is sufficient to decrease action potential firing. (D) Left, inactive and active lever presses and heroin infusions during the final 2 days of FR1 and final 3 days of trial‐based self‐administration sessions. During the final 3 days of trial‐based self‐administration, these measures did not differ between eNpHR3.0 and eYFP groups and were similar in females (top middle) and males (bottom middle). Due to the scale of the y‐axis and lack of differences between groups, the eYFP group's trial‐based self‐administration data are obscured by the eNpHR3.0 group data. Right, the percent of trials with an active lever press during the final 3 days of self‐administration also did not differ between eNpHR3.0 and eYFP groups. Individual data values are shown for females (yellow) and males (cyan). (E) Postlever press IL inhibition during the first 5 days of extinction had no effect on active lever pressing during these sessions, nor on the subsequent 7 days of extinction without laser illumination. Similar results were observed in females (top right) and males (bottom right).

In this experiment, the IL was inhibited for 20 s immediately following an unreinforced lever press during the first 5 days of extinction (Figure 1A,B). Figure 1C shows electrophysiological confirmation of functional eNpHR3.0 expression in the IL during whole‐cell recordings. A decrease in action potential firing during LED illumination confirms eNpHR3.0‐mediated inhibition. Figure 1D shows self‐administration measures across the final 2 days of FR1 self‐administration and the final 3 days of trial‐based self‐administration. Because of lever retraction during the trial‐based procedures, active lever presses were equal to the number of heroin infusions and were not shown or analysed separately. There were no pre‐existing differences in the final 3 days of trial‐based self‐administration data between eNpHR3.0 and eYFP groups, although there was a nonsignificant trend toward an interaction effect between day and group for heroin infusions (Table 1). Additionally, there was no difference in the average percent of trials with an active lever press during the final 3 days of trial‐based self‐administration between groups (*t*(30.70) = 0.81, *p* = 0.42). Figure 1E shows active lever presses across extinction sessions (inactive lever presses did not significantly differ between groups and are not shown). Statistics for females and males are shown in Table 2. Analysis of active lever presses during the first 5 days of extinction with laser illumination revealed a main effect of day (*F*_1.97,61.05_ = 19.09, *p* < 0.0001), but no main effect of inhibition (*F*_1,31_ = 0.20, *p* = 0.66) or interaction (*F*_4,124_ = 0.94, *p* = 0.44). Similarly, analysis of active lever presses during the next 7 days of extinction without laser illumination revealed a main effect of day (*F*_3.73,115.7_ = 16.78, *p* < 0.0001), but no main effect of inhibition (*F*_1,31_ = 0.32, *p* = 0.58) or interaction (*F*_6,186_ = 1.35, *p* = 0.24). Thus, postlever press IL inhibition had no effect on active lever pressing during the first 5 days of extinction, nor on retention of extinction learning as measured by active lever pressing during the next 7 days of extinction without inhibition.

**TABLE 1:** Statistics for the final 3 days of self‐administration from each experiment using two‐way repeated measures ANOVA.

Measure	Effect	Experiment 1	Experiment 2	Experiment 3
Inactive presses	Group	*F*_1,31_ = 0.03, *p* = 0.87	*F*_1,19_ = 0.56, *p* = 0.46	*F*_1,23_ = 0.56, *p* = 0.46
	Day	*F*_1.07,33.04_ = 1.52, *p* = 0.23	*F*_1.29,24.43_ = 0.36, *p* = 0.61	*F*_1.36,31.26_ = 1.68, *p* = 0.21
	Interaction	*F*_2,62_ = 0.25, *p* = 0.78	*F*_2,38_ = 1.38, *p* = 0.26	*F*_2,46_ = 0.44, *p* = 0.64
Infusions	Group	*F*_1,31_ = 1.24, *p* = 0.27	*F*_1,19_ = 0.75, *p* = 0.40	*F*_1,23_ = 0.36, *p* = 0.55
	Day	*F*_1.80,55.92_ = 5.00, *p* = 0.01	*F*_1.55,29.42_ = 4.68, *p* = 0.02	*F*_1.58,36.38_ = 2.12, *p* = 0.14
	Interaction	*F*_2,62_ = 2.49, *p* = 0.09	*F*_2,38_ = 0.56, *p* = 0.57	*F*_2,46_ = 3.33, *p* = 0.04

**TABLE 2:** Female and male statistics for active lever presses during extinction from Experiment 1 (IL inhibition) using two‐way repeated measures ANOVA.

Behaviour	Effect	Female	Male
Extinction with laser	Group	*F*_1,13_ = 0.08, *p* = 0.78	*F*_1,16_ = 1.45, *p* = 0.25
	Day	*F*_1.84,23.88_ = 24.47, *p* < 0.0001	*F*_4,64_ = 5.41, *p* < 0.001
	Interaction	*F*_4,52_ = 0.83, *p* = 0.51	*F*_4,64_ = 0.27, *p* = 0.90
Extinction without laser	Group	*F*_1,13_ = 0.36, *p* = 0.56	*F*_1,16_ = 2.35, *p* = 0.14
	Day	*F*_2.90,37.70_ = 12.45, *p* < 0.0001	*F*_6,96_ = 7.52, *p* < 0.0001
	Interaction	*F*_6,78_ = 1.65, *p* = 0.14	*F*_6,96_ = 1.39, *p* = 0.23

### 3.2 Experiment 2

**FIGURE 2:**
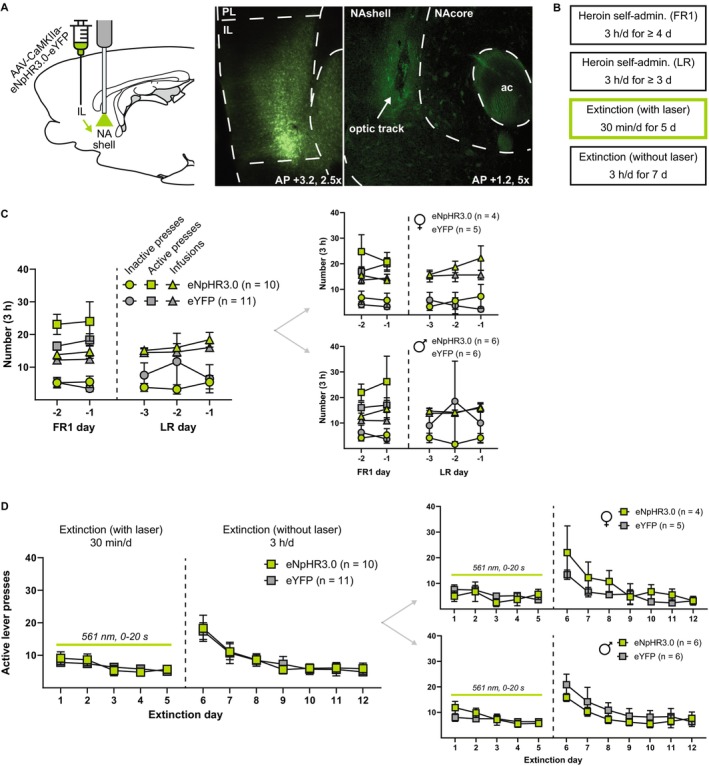
No effect of postlever press IL‐NAshell inhibition on heroin extinction learning. (A) Left, a viral vector containing either the inhibitory opsin eNpHR3.0 or an eYFP control was injected into the IL, and optical fibres were implanted above the NAshell to target IL terminals. Right, representative fluorescent images depicting virus expression in IL cell bodies and virus expression in IL terminals in the NAshell where the optical fibre terminates. (B) Experimental timeline. After recovering from surgery, rats were trained on 3‐h FR1 heroin self‐administration, followed by 3‐h FR1 sessions with lever retraction (LR) wherein an active lever press resulted in a heroin infusion, light and tone cues and 20‐s active lever retraction, but levers were otherwise available throughout the duration of the session. Self‐administration was followed by 5 days of 30‐min extinction sessions with laser illumination, wherein each active lever press resulted in 20‐s active lever retraction and laser illumination for the duration of the lever retraction. Rats then underwent 7 days of 3‐h extinction sessions without laser illumination to assess the retention of extinction learning. (C) Left, inactive and active lever presses and heroin infusions during the final 2 days of FR1 self‐administration and final 3 days of FR1 sessions with lever retraction. During the final 3 days of FR1 sessions with lever retraction, these measures did not differ between eNpHR3.0 and eYFP groups and were similar in females (top right) and males (bottom right). (D) Postlever press inhibition of the IL‐NAshell pathway during the first 5 days of extinction had no effect on active lever pressing during these sessions, nor on the subsequent 7 days of extinction without laser illumination. Similar results were observed in females (top right) and males (bottom right).

In this experiment, the IL‐NAshell pathway was inhibited for 20 s immediately following an unreinforced lever press during the first 5 days of extinction (Figure 2A,B). Figure 2C shows self‐administration measures across the final 2 days of FR1 self‐administration and the final 3 days of FR1 self‐administration with lever retraction. During FR1 self‐administration with lever retraction, active lever presses were equal to the number of heroin infusions and were not shown or analysed separately. There were no pre‐existing differences in the final 3 days of self‐administration with lever retraction data between eNpHR3.0 and eYFP groups (Table 1). Figure 2D shows active lever presses across extinction sessions (inactive lever presses did not significantly differ between groups and are not shown). Statistics for females and males are shown in Table 3. Analysis of active lever presses during the first 5 days of extinction with laser illumination revealed a main effect of day (*F*_2.80,53.21_ = 5.63, *p* < 0.01), but no main effect of inhibition (*F*_1,29_ = 0.02, *p* = 0.88) or interaction (*F*_4,76_ = 0.81, *p* = 0.52). Similarly, analysis of active lever presses during the next 7 days of extinction without laser illumination revealed a main effect of day (*F*_3.23,61.35_ = 20.22, *p* < 0.0001), but no main effect of inhibition (*F*_1,19_ = 0.01, *p* = 0.94) or interaction (*F*_6,114_ = 0.19, *p* = 0.98). Thus, postlever press IL‐NAshell inhibition had no effect on active lever pressing during the first 5 days of extinction, nor on retention of extinction learning as measured by active lever pressing during the next 7 days of extinction without inhibition.

**TABLE 3:** Female and male statistics for active lever presses during extinction from Experiment 2 (IL‐NAshell inhibition) using two‐way repeated measures ANOVA.

Behaviour	Effect	Female	Male
Extinction with laser	Group	*F*_1,7_ = 0.25, *p* = 0.63	*F*_1,10_ = 0.31, *p* = 0.59
	Day	*F*_2.15,15.06_ = 2.15, *p* = 0.15	*F*_2.27,22.69_ = 4.15, *p* = 0.03
	Interaction	*F*_4,28_ = 1.06, *p* = 0.39	*F*_4,40_ = 1.53, *p* = 0.21
Extinction without laser	Group	*F*_1,7_ = 0.81, *p* = 0.40	*F*_1,10_ = 0.62, *p* = 0.45
	Day	*F*_1.74,12.20_ = 9.15, *p* < 0.01	*F*_3.10,31.01_ = 11.54, *p* < 0.0001
	Interaction	*F*_6,42_ = 1.00, *p* = 0.44	*F*_6,60_ = 0.66, *p* = 0.68

### 3.3 Experiment 3

**FIGURE 3:**
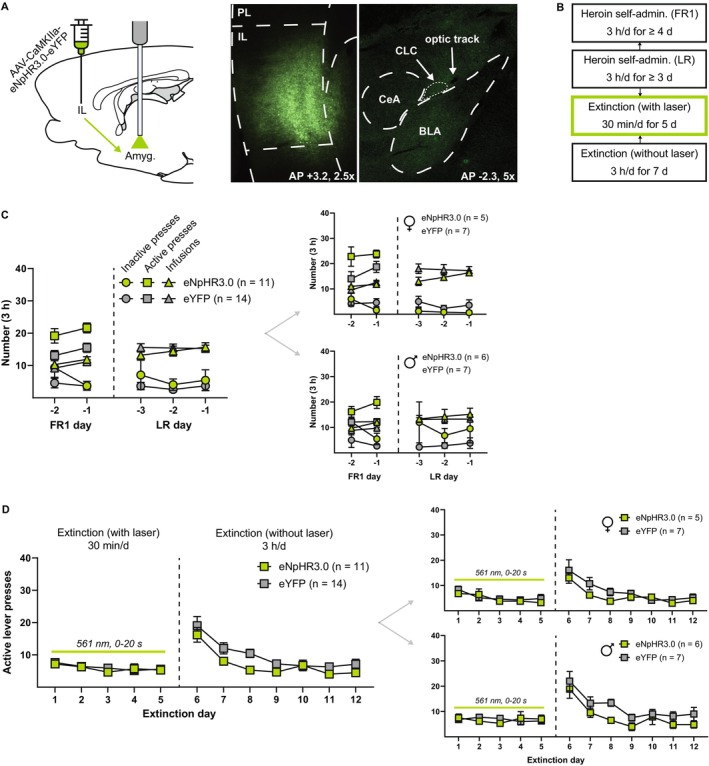
No effect of postlever press IL‐amygdala inhibition on heroin extinction learning. (A) Left, a viral vector containing either the inhibitory opsin eNpHR3.0 or an eYFP control was injected into the IL, and optical fibres were implanted above the amygdala to target IL terminals in the capsular cells of the central nucleus (CLC) and basolateral amygdala (BLA). Right, representative fluorescent images depicting virus expression in IL cell bodies and virus expression in IL terminals in the amygdala where the optical fibre terminates. (B) Experimental timeline. After recovering from surgery, rats were trained on 3‐h FR1 heroin self‐administration, followed by 3‐h FR1 sessions with lever retraction (LR) wherein an active lever press resulted in a heroin infusion, light and tone cues and 20‐s active lever retraction, but levers were otherwise available throughout the duration of the session. Self‐administration was followed by 5 days of 30‐min extinction sessions with laser illumination, wherein each active lever press resulted in 20‐s active lever retraction and laser illumination for the duration of the lever retraction. Rats then underwent 7 days of 3‐h extinction sessions without laser illumination to assess the retention of extinction learning. (C) Inactive and active lever presses and heroin infusions during the final 2 days of FR1 self‐administration and final 3 days of FR1 sessions with lever retraction. During the final 3 days of FR1 sessions with lever retraction, these measures did not differ between eNpHR3.0 and eYFP groups, although there was a significant interaction effect between day and group for heroin infusions. Measures were similar in females (top right) and males (bottom right). (D) Postlever press inhibition of the IL‐amygdala pathway during the first 5 days of extinction had no effect on active lever pressing during these sessions, nor on the subsequent 7 days of extinction without laser illumination. Similar results were observed in females (top right) and males (bottom right).

In this experiment, the IL‐amygdala pathway was inhibited for 20 s immediately following an unreinforced lever press during the first 5 days of extinction (Figure 3A,B). Figure 3C shows self‐administration measures across the final 2 days of FR1 self‐administration and the final 3 days of FR1 self‐administration with lever retraction. During FR1 self‐administration with lever retraction, active lever presses were equal to the number of heroin infusions and were not shown or analysed separately. There were no pre‐existing differences in the final 3 days of self‐administration with lever retraction data between eNpHR3.0 and eYFP groups, although there was a significant interaction effect between day and group for heroin infusions (Table 1). Figure 3D shows active lever presses across extinction sessions (inactive lever presses did not significantly differ between groups and are not shown). Statistics for females and males are shown in Table 4. Analysis of active lever presses during the first 5 days of extinction with laser illumination revealed a trend toward a main effect of day (*F*_3.46,79.56_ = 2.43, *p* = 0.06), but no main effect of inhibition (*F*_1,23_ = 0.15, *p* = 0.70) or interaction (*F*_4,92_ = 0.33, *p* = 0.85). Similarly, analysis of active lever presses during the next 7 days of extinction without laser illumination revealed a main effect of day (*F*_2.71,62.24_ = 31.08, *p* < 0.0001), but no main effect of inhibition (*F*_1,23_ = 2.902, *p* = 0.10) or interaction (*F*_6,138_ = 1.094, *p* = 0.37). Thus, postlever press IL‐NAshell inhibition had no effect on active lever pressing during the first 5 days of extinction, nor on retention of extinction learning as measured by active lever pressing during the next 7 days of extinction without inhibition.

**TABLE 4:** Female and male statistics for active lever presses during extinction from Experiment 3 (IL‐amygdala inhibition) using two‐way repeated measures ANOVA.

Behaviour	Effect	Female	Male
Extinction with laser	Group	*F*_1,10_ = 0.30, *p* = 0.60	*F*_1,11_ = 0.02, *p* = 0.89
	Day	*F*_2.56,25.56_ = 4.55, *p* = 0.01	*F*_2.54,27.89_ = 0.14, *p* = 0.91
	Interaction	*F*_4,40_ = 0.65, *p* = 0.63	*F*_4,40_ = 0.65, *p* = 0.63
Extinction without laser	Group	*F*_1,10_ = 1.11, *p* = 0.32	*F*_1,11_ = 2.48, *p* = 0.14
	Day	*F*_2.01,20.17_ = 12.58, *p* < 0.001	*F*_2.67,29.33_ = 18.71, *p* < 0.0001
	Interaction	*F*_6,60_ = 0.84, *p* = 0.55	*F*_6,66_ = 0.54, *p* = 0.78

### 3.4 Sex Differences in Heroin Self‐Administration

**FIGURE 4:**
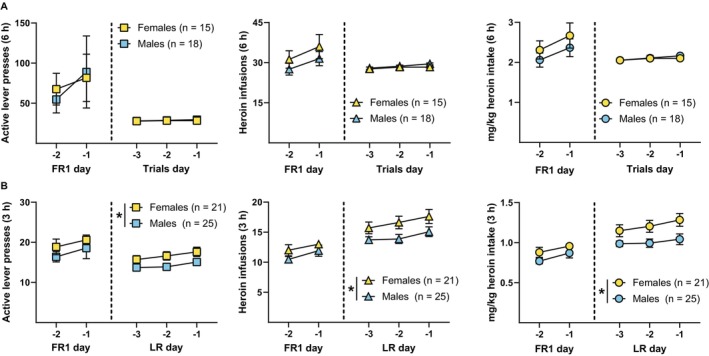
Sex differences in 3 h, but not 6 h, heroin self‐administration. (A) Daily active lever presses (left), heroin infusions (middle) and mg/kg heroin intake (right) across the final 2 days of FR1 and final 3 days of trial‐based 6‐h self‐administration sessions for females and males, collapsed across eNpHR3.0 and eYFP groups from Experiment 1. There were no significant differences between females and males in these measures during the final 3 days of 6‐h trial‐based self‐administration. (B) Daily active lever presses (left), heroin infusions (middle) and mg/kg heroin intake (right) across the final 2 days of FR1 and final 3 days of FR1 with lever retraction (LR) 3‐h self‐administration sessions for females and males, collapsed across eNpHR3.0 and eYFP groups from Experiments 2 and 3. Females had significantly more heroin infusions and mg/kg heroin intake than males during the final 3 days of 3‐h FR1 self‐administration with lever retraction. *p < 0.05.

We also conducted ancillary analyses of self‐administration data to determine whether there were any sex differences in 6‐ or 3‐h heroin self‐administration. Figure 4A shows daily active lever presses, heroin infusions and mg/kg heroin intake across both the final 2 days of FR1 and final 3 days of trial‐based 6‐h self‐administration sessions for females and males, collapsed across eNpHR3.0 and eYFP groups from Experiment 1. Due to lever retraction procedures, active lever presses were equal to the number of heroin infusions during the final 3 days of self‐administration and were not analysed separately. Analysis of heroin infusions during the final 3 days of trial‐based self‐administration revealed a main effect of day (*F*_1.86,57.77_ = 4.40, *p* = 0.02), but no main effect of sex (*F*_1,31_ = 1.60, *p* = 0.21), or interaction (*F*_2,62_ = 0.94, *p* = 0.40). Similarly, analysis of mg/kg heroin intake during these days showed a main effect of day (*F*_1.93,59.69_ = 3.35, *p* = 0.04), but no main effect of sex (*F*_1,31_ = 0.13, *p* = 0.72), or interaction (*F*_2,62_ = 0.56, *p* = 0.58). Figure 4B shows daily active lever presses, heroin infusions and mg/kg heroin intake across both the final 2 days of FR1 and final 3 days of FR1 with lever retraction for females and males, collapsed across eNpHR3.0 and eYFP groups from Experiments 2 and 3. Analysis of heroin infusions during the final 3 days of self‐administration with lever retraction revealed a significant main effect of day (*F*_1.70,74.82_ = 5.68, *p* < 0.01) and sex (*F*_1,44_ = 4.83, *p* = 0.03), with females receiving significantly more infusions than males, but no significant interaction (*F*_2,88_ = 0.30, *p* = 0.74). Similarly, analysis of mg/kg heroin intake during these days revealed a trend toward a main effect of day (*F*_1.64,72.09_ = 3.26, *p* = 0.05) and a main effect of sex (*F*_1,44_ = 6.66, *p* = 0.01), with females taking significantly more heroin by body weight than males, but no significant interaction (*F*_2,88_ = 0.55, *p* = 0.58).

## 4 Discussion

The present findings indicate that postlever press optogenetic inhibition of IL cell bodies, or the IL‐NAshell or IL‐amygdala pathways, has no effect on the extinction of heroin seeking, as assessed by lever pressing during extinction sessions both with and without inhibition. This contrasts with our prior work with cocaine [2, 3], wherein the same manipulations impaired the extinction of cocaine seeking. These results suggest that the encoding of heroin extinction does not require the same infralimbic mechanisms that are important for cocaine extinction encoding.

### 4.1 Extinction Mechanisms Are Not Universal

Although our previous work found that IL activity is important for encoding extinction learning for cocaine seeking, the present study suggests that this finding does not generalize across all drug classes. This is somewhat surprising given evidence that IL‐amygdala inhibition impairs extinction of both cocaine seeking [3] and conditioned fear [15], indicating that some of these infralimbic mechanisms generalize to behavioural extinction outside drug self‐administration. Moreover, other evidence suggests that inactivating an extinction ensemble in the IL increases responding for a highly valuable food reward [26], although our prior work found no effect of IL inhibition on extinction of standard food seeking [2]. Possibly the different mechanisms of action for opioids versus psychostimulants like cocaine [27] may account for the discrepancy between this work and our previous studies [2, 3]. Notably, evidence suggests divergent regulation of heroin vs. cocaine seeking by the IL [18], which may reflect the degree to which hedonic vs. homeostatic mechanisms regulate drug seeking [28, 29]. Another possibility is that the extinction of heroin seeking recruits a larger network of extinction circuitry that is sufficient for encoding extinction learning even when the IL and its projections are inhibited. This idea could explain why IL activation enhances heroin extinction learning [9], but IL inhibition did not impair the extinction of heroin seeking in the current work.

Another possible explanation for the present results is that heroin‐extinction encoding involves a critical window of IL activity that is not captured in the first 20‐s postlever press. Limited evidence indicates that the response dynamics of midbrain dopamine neurons and serotonin neurons to a heroin infusion can be slower than to a cocaine infusion, depending on dose [30], whereas dopamine levels in the brain peak within 10–20 s of an intravenous cocaine infusion [31]. Nonetheless, as an opioid, heroin also directly and rapidly activates opioid receptors that can be reinforcing independent of the dopamine system [32]. Moreover, in the present study, the conditioned reinforcers were presented for 5 s immediately following an active lever press during self‐administration but not during extinction. Thus, it seems likely that at least a portion of the prediction error during extinction occurs within the 20‐s window. Nonetheless, the timing of IL inhibition relative to the prediction error signal is an important consideration when comparing results across drug types. Struik et al. [33] found that 6 s of postpress IL inhibition had no effect on cued extinction of nicotine seeking; however, in that study, lever pressing during extinction still produced the nicotine‐associated cues, which likely would have delayed the prediction error signal outside the 6‐s inhibition window. A number of methodological differences make it difficult to compare that finding to the current results, but it raises further questions about the role of the IL in extinction learning across drugs of abuse.

Nonetheless, a mechanism necessary for the extinction of opioid seeking remains elusive. Recent work from our laboratory indicates that anterior insular cortex (aIC) activity inhibits cued heroin seeking after extinction learning, but not after prolonged withdrawal without additional contingency learning, suggesting an extinction‐dependent role for this region in inhibiting heroin seeking [34]. Interestingly, this contrasts with a role for the aIC in promoting cocaine seeking after extinction [35]. Whether opioid extinction learning requires the aIC remains uninvestigated and would be a promising target for future work.

### 4.2 IL‐NAshell Regulation of Heroin Seeking

Heinsbroek et al. [6] found that chemogenetic inactivation of the IL‐NAshell pathway selectively increased heroin choice and relapse in a procedure wherein rats learned to make distinct responses for heroin and food rewards, suggesting that this pathway exerts inhibitory control over heroin seeking after new contingency learning. The difference with our present findings could be explained by the timing of the pathway inhibition (during the expression of drug seeking vs. during encoding of the new extinction contingency) or by distinct mechanisms for suppressing heroin seeking in favour of food reward vs. in response to unrewarded lever presses during extinction. A review of the neural mechanisms underlying drug relapse and craving after voluntary abstinence reveals that these mechanisms are often different from those involved after extinction learning [36]. Indeed, Bossert et al. [19] found that disconnection of the IL‐NAshell pathway decreased heroin seeking after extinction, indicating that the direction in which this pathway regulates heroin seeking is highly dependent on the type of contingency learning. Because of this, we cannot exclude the possibility that the low number of lever presses during extinction in the present study created a floor effect, potentially obscuring an *enhancement* of extinction learning by postpress IL‐NAshell inhibition.

### 4.3 Sex Differences in Heroin Self‐Administration

Analyses of self‐administration data revealed that females self‐administered significantly more heroin than males during 3‐h, but not 6‐h, sessions. The lack of sex differences in 6‐h heroin self‐administration is consistent with prior work from our laboratory [34, 37] and some others [24, 38], although Lynch and Carroll [24] found that female rats acquire 6‐h heroin self‐administration more quickly than males. Conversely, George et al. [39] found that female rats self‐administered significantly more heroin than males in both 3‐ and 6‐h sessions. Cicero et al. [23] found that females self‐administered significantly more heroin than males on an FR4 schedule in 4‐h sessions, yet Stewart et al. [25] found no sex differences when rats self‐administered heroin in four 3‐h sessions per day. Taken together, it appears that female rats self‐administer more heroin than males under certain conditions, but the variety of methodological parameters used makes it difficult to draw conclusions about what these conditions might be.

## 5 Conclusion

The present findings, together with our prior work with cocaine, add to the evidence of differential IL regulation of opioid vs. psychostimulant seeking, indicating that mechanisms of extinction are also drug‐type dependent. Although it remains unclear under which conditions the IL and its projections to the NAshell and amygdala regulate heroin seeking, 20‐s inhibition of any of this circuitry following an unreinforced lever press does not disrupt a critical encoding period for heroin extinction. These results highlight the need for more mechanistic comparisons across drug types and raise further questions about the circuitry that exerts inhibitory control over opioid seeking.

## Author Contributions

M.S.M., K.E.N. and R.T.L. contributed to the conception and design of Experiments 1, 2 and 3. K.E.N. contributed to data acquisition for Experiment 1. M.S.M. contributed to data acquisition and analysis for Experiments 1, 2 and 3, as well as drafting this work. S.C.G. and J.A.W. contributed to the design and acquisition of the electrophysiological verification experiment. All authors contributed to the article and approved the submitted version.

## Conflicts of Interest

The authors declare no conflicts of interest.

## Supporting information


**Data S1:** Supporting information.

## Data Availability

The data that support the findings of this study are available in the Supporting Information of this article.
